# The Dental Protective Association

**Published:** 1891-11

**Authors:** 


### Editorial.

### THE DENTAL PROTECTIVE ASSOCIATION.

It may seem to some an anomaly that dentistry should need protection more than any other kindred profession, or that it should require an association with large capital to protect it from the encroachments of monopolies working under the authority of the seal of the United States.

The experience of the dental profession in this part of the world in the operation of patent-laws has not been a pleasant one. Indeed, the history of the past, familiar to all, is replete with injury to individuals, and, in the broader sense, has been a continued hinderance to true progress.

Patent laws have, unquestionably, been a great incentive to improvement, but they have equally stimulated the grasping spirit of capital. They have quickened the inspiration for development, and have, by the power conferred, destroyed many original and valuable improvements, that others might have no competition in the marts of trade. This crushing-out process is not peculiar to any one line of business, but seems to be a necessary outgrowth of the laws adopted for the protection of the inventor, and has assumed proportions in direct ratio to the value of certain products.

The Dental Protective Association, which in one aspect seems unprofessional, has been the growth of necessity, and its development is one of the marvels of the past decade. We believe it to be peculiar to dentistry, as we know of no other similar organization so thoroughly arranged or so completely under control as this has been demonstrated to be. When it is considered that this work has been mainly accomplished through the indomitable energy of one man, Dr. J. N. Crouse, of Chicago, the surprise is increased.

Revolutions develop men and measures, "new occasions teach new duties," and it has been left for our profession, untrammelled with precedents, to mark a path for others to follow.

The Association is not in opposition to law, but in harmony with it. It is organized to test the rights of individuals, and when the last court has been reached, and the decision given, it closes further contest in any given case. It simply proposes to perform a duty not possible for individuals, and says to the combinations, in emphatic language, "Your claims are not recognized until the final record has been made, and it is your duty to make these good, as it is ours to contest them."

The results thus far obtained have been most satisfactory, as the reports of the organization have amply demonstrated; and while the legal work will be continued, the future seems to promise an enlarged sphere of action possibly not contemplated by its originators.

The dental profession, like all other bodies of a similar character, is to-day widely scattered over this continent. While it numbers fifteen thousand, and constantly increasing, the membership in the organization does not, as yet, comprise a proper proportion of this large body. Every individual should have his name on the books,—not solely for self-protection, but that he may come directly in touch with his fellow-practitioners throughout this broad domain. There is something inspiriting in the feeling of being one with a large, active profession, and, however isolated the individual may be, the pulsations of the great body will be felt, and just in that proportion will each one be strengthened and elevated.

This is the broad view of the work, and it is by no means the least. The possibilities of an organization such as this are not easily to be realized at the present. We regard it as one of the active agents in elevating dentistry, binding the individual members together as one man in the progressive strides towards professional unity.

The legal aspects are now the most important. They naturally appeal to the self-interest side of every practitioner; but while they do this, let the higher thought be not forgotten,—that the ultimate tendency of this organization is to lead to a union of effort in all that is uplifting and to that which marks a broader conception of duty.

